# Influence of Initial pH Value on the Adsorption of Reactive Black 5 Dye on Powdered Activated Carbon: Kinetics, Mechanisms, and Thermodynamics

**DOI:** 10.3390/molecules27041349

**Published:** 2022-02-16

**Authors:** Branka Vojnović, Mario Cetina, Petra Franjković, Ana Sutlović

**Affiliations:** 1 Department of Applied Chemistry, Faculty of Textile Technology, University of Zagreb, Prilaz baruna Filipovića 28a, 10000 Zagreb, Croatia; branka.vojnovic@ttf.unizg.hr (B.V.); petra@franjkovic.com (P.F.); 2 Department of Textile Chemistry and Ecology, Faculty of Textile Technology, University of Zagreb, Savska c. 16/9, 10000 Zagreb, Croatia; ana.sutlovic@ttf.unizg.hr

**Keywords:** adsorption, activated carbon, Reactive Black 5, kinetics, thermodynamics

## Abstract

The aim of this work was to investigate the influence of initial pH value (pH_0_) on the isothermal adsorption of Reactive Black 5 (RB5) dye on commercial powdered activated carbon. Four initial pH values were chosen for this experiment: pH_0_ = 2.00, 4.00, 8.00, and 10.00. In order to investigate the mechanism of adsorption kinetic, studies have been performed using pseudo-first-order and pseudo-second-order kinetic models as well as an intraparticle diffusion model. In addition, thermodynamic parameters of adsorption were determined for pH_0_ = 4.00. Results of this research showed that the initial pH value significantly influences the adsorption of RB5 dye onto activated carbon. The highest adsorption capacities (*q_e_*) and efficiencies of decolouration were observed for initial pH values of pH_0_ = 2.00 (*q_e_* = 246.0 mg g^−1^) and 10.00 (*q_e_* = 239.1 mg g^−1^) due to strong electrostatic interactions and attractive π···π interactions, respectively. It was also shown that the adsorption of RB5 dye on activated carbon at all initial pH values is kinetically controlled, assuming a pseudo-second-order model, and that intraparticle diffusion is not the only process that influences on the adsorption rate.

## 1. Introduction

The textile industry has a positive impact on economic development around the world, because of constantly growing demands for textile products. The main environmental textile industry problem is the fact that its intensive development causes an increased use of resources, especially in terms of water consumption, and releases highly contaminated wastewaters with a wide range of unprocessed harmful and toxic chemicals. A further problem is that some less harmful chemicals are used in huge quantities and therefore also produce a lot of waste [1,2]. Wet treatment textile industry processes (e.g., finishing, dyeing, printing etc.) are the main sources of toxic emissions into the environment—water, air, and soil. Many textile-processing operations generate large amounts of pollutants and pose a threat to the environment if they are not appropriately treated. One of the most studied textile industry process in the context of wastewaters is dyeing. Even very low concentrations of dyes in wastewaters are noticeable, which makes water aesthetically and ecologically unacceptable. At the same time, colour reduces the transparency of the water and creates the impression of a high degree of pollution. The presence of water colouring causes less penetration of sunlight into the depths of the water, which leads to the disruption of the process of photosynthesis and prevents animals’ orientation. In addition, some dyes are toxic, while auxiliaries such as carriers, metals, salts, etc. used in the dying process also contribute to the water pollution. Therefore, the wastewater stream from the textile dyeing process besides unutilized dyes (about 8−20% of the total pollution load due to incomplete dye exhaustion) contains other auxiliary chemicals. Textile dyeing wastewaters also contain high pH values, high salt concentrations, high total suspended solids, high temperatures, and high chemical oxygen demand (COD) and biochemical oxygen demand (BOD) values [3,4].

There are more than 100,000 commercially available dyes, and more than 700,000 tons of dyes are produced annually. As a result of their benefits, such as vibrant colours, high colourfastness, and simplicity of application, reactive dyes are one of the most often used dyes [4]. However, the removal of reactive dyes from wastewaters is generally difficult. They are usually resistant to aerobic digestion and stable to light, heat, and oxidising agents. Due to their relatively poor biodegradation in aerobic circumstances (particularly those containing azo groups) and their increased use, they had an impact on traditional methods for treating textile wastewaters [4,5]. Therefore, finding the appropriate method for the treatment of a particular type of textile wastewater is very important. For this purpose, wastewater is collected and subjected to physical, chemical, and/or biological treatment processes before being returned to the environment.

Many different methods and techniques have been reported for the removal and/or decolourisation of high soluble reactive dyes from wastewaters: coagulation and flocculation, chemical oxidation, biological treatment, membrane separation, reverse osmosis, etc. [4,6,7,8,9]. Chemical and biological methods could be very effective, but they require specialised equipment; they are energy intensive, use an excess of chemicals, and may produce large amounts of solid waste or could generate environmentally unsuitable by-products. The high solubility of reactive dyes causes a relatively poor colour removal efficiency by the coagulation and flocculation method, so that they have a limited application [7]. On the other hand, physical and physicochemical methods (adsorption, ion exchange, membrane filtration, etc.) have proven to be very effective for the removal of reactive dyes. Adsorption is one of the best treatment methods due to its flexibility, simplicity of design, industrial application, and sensitivity to toxic pollutants [4,8,10]. Many studies on the development and practical application of different effective adsorbents for reactive dyes removal, including Reactive Black 5 dye discussed in this paper, have been reported. These studies and recent reviews include adsorbents such as natural and by-product adsorbents [11,12,13], surface-modified natural adsorbents [14], nanostructures [15,16], chitosan-based adsorbents [10,17,18], aminosilosan functionalised silica [19], and mixed silica-alumina oxides [20,21]. Modern adsorbents, such as nanostructures, are highly effective for Reactive Black 5 removal, but they are expensive, and therefore, it is essential to regenerate and reuse them for the water treatment [15,16]. The advantages of natural and by-product adsorbents is their low cost, easy accessibility, and usage of waste materials such as agricultural crop or industrial residues as a source for activated carbon production [11,12,13,22]. Activated carbon is one of the most popular adsorbents used for wastewater treatment due to its adsorption efficiency, great capacity, and very high quality of purified water [22,23,24]. In addition, adsorption on activated carbon generally does not result in the formation of harmful substances, and it can be used for the control of precursors that may form toxic compounds [25].

It is known that the adsorption process significantly depends on several factors, including temperature, optimal dose of adsorbent, contact time, initial dye concentration, and pH value. Therefore, in this study, the adsorption of Reactive Black 5 (RB5) onto commercial powdered activated carbon at different initial pH values was examined. Most adsorption studies have been conducted on acidic and neutral media [10]. On the other hand, we performed adsorption experiments in a wider range of initial pH values, from pH_0_ = 2.00 to pH_0_ = 10.00. It should be pointed out that the adsorption of RB5 dye on commercial powdered activated carbon at high pH values, such as pH_0_ = 10.00, which is used in real RB5 dye industrial application (e.g., for cellulose fibres dying), is rarely described in the literature. We monitored pH value during the whole adsorption time, which helped us to presume adsorption mechanisms at different initial pH values. The kinetics and thermodynamics for this adsorption system were also investigated.

## 2. Results and Discussion

### 2.1. Effect of Initial pH Value on Adsorption Process

**Figure 1:**
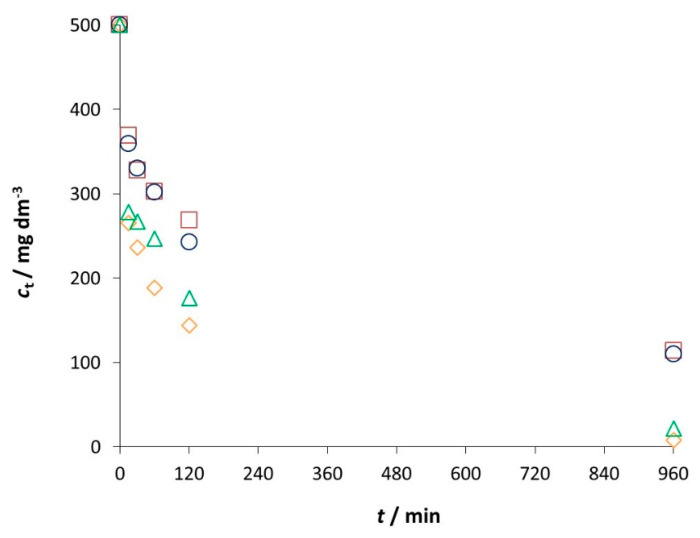
Effect of initial pH values on the RB5 dye concentration in the liquid phase (*c_t_*) after appropriate time of adsorption (*t*) (**◇** pH_0_ = 2.00; **☐** pH_0_ = 4.00; **O** pH_0_ = 8.00; **△** pH_0_ = 10.00).

The objective of this work was to evaluate the influence of initial pH value (pH_0_) on the treatment of dye-rich textile wastewaters by the adsorption process. For this study, we chose an RB5 dye concentration of *c*_0_ = 500 mg dm^−^^3^, which is a possible dye concentration in textile wastewaters. The amounts of adsorbed dye at time *t* (*q_t_*) for different initial pH values are given in Table 1, while the liquid phase dye concentrations (*c_t_*) during adsorption process are presented in Figure 1.

The data in Table 1 show that the initial pH value significantly influences adsorption capacity, i.e., the amount of adsorbed dye at equilibrium (*q_e_*). The highest adsorption capacities were observed for initial pH values of pH_0_ = 2.00 and 10.00 (*q_e_* = 246.0 and 239.1 mg g^−^^1^, respectively), while values for pH_0_ = 4.00 and 8.00 are much lower (*q_e_* < 200 mg g^−^^1^). From Figure 1, it is obvious that dye concentration in the liquid phase decreases very fast. The plots can be approximately divided into several regions: very fast initial adsorption, then a milder and gradual decrease of dye concentration, which then reaches the equilibrium state. In this figure, one can also see that dye concentrations in the liquid phase for pH_0_ = 4.00 and 8.00 are approximately equal for all contact times, with the exception for a contact time of 2 h.

To calculate the efficiency of decolouration (*E_d_*) for all initial pH values, we compared with results obtained using the same procedure of adsorption experiment and applying the same conditions (dye concentration, mass of adsorbent, and temperature) but without changing the pH value before adsorption [26] (Table 2).

**Scheme 1:**
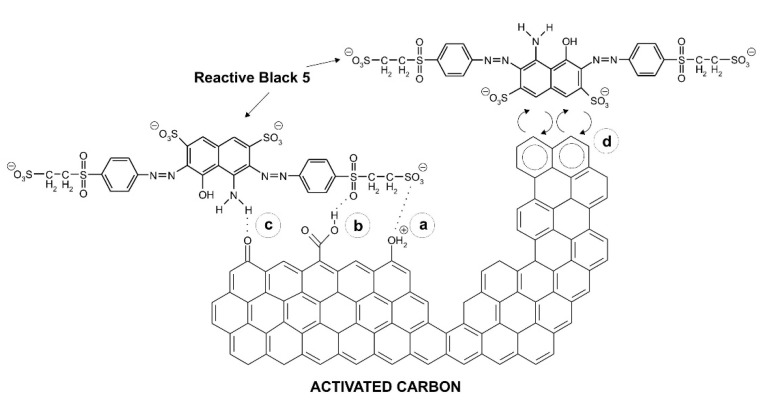
Schematic representation of some interactions between activated carbon (AC) and RB5 anion: (**a**) electrostatic interaction between positively charged protonated hydroxyl group of AC and negatively charged sulfonic RB5 group, (**b**) O–H⋅⋅⋅O hydrogen bonding between carboxyl group of AC and sulfonyl group of RB5, (**c**) N–H⋅⋅⋅O hydrogen bonding between amino group of RB5 and carbonyl group of AC, (**d**) π⋅⋅⋅π interaction between naphthalene ring of RB5 and graphene layers of AC.

As already noticed for adsorption capacities, the results show that the biggest values of *E*_d_ were observed for pH_0_ = 2.00 and pH_0_ = 10.00, which at equilibrium amount to 98.4% and 95.6%, respectively. After 15 min, *E*_d_ values already reached ca. 45% and are almost twice as big as those for pH_0_ = 4.00 and pH_0_ = 8.00. At all time intervals, the *E*_d_ values follow the sequence: *E_d_* (pH_0_ = 2.00) > *E_d_* (pH_0_ = 10.00) > *E_d_* (pH_0_ = 4.00 ≈ pH_0_ = 8.00). In addition, the values obtained for pH_0_ = 4.00 are approximately equal to those for pH_0_ = 4.83, when the pH value before the adsorption experiment was not changed [26]. The fact that *E*_d_ values are the biggest for pH_0_ = 2.00 can be explained by the activated carbon surface charge and interactions present between activated carbon and RB5 dye, which dissociate on coloured anion and sodium ions. If the pH value is less than the activated carbon point of zero charge (pH_PZC_), its surface is positively charged and adsorbs the coloured anions due to strong electrostatic attraction. The lower the pH value relative to the pH_PZC_, the stronger these attractions. It has been previously reported that the pH_PZC_ values for commercial powdered activated carbons range from 6.50 to 7.33 [27,28,29,30]. Such values support the fact that extremely strong adsorption at pH_0_ = 2.00 could be attributed to very strong electrostatic interactions between the surface of the activated carbon and dye anions. The protonated groups of activated carbon are mainly carboxylic, hydroxyl, and chromenic. An example of such interaction between positively charged protonated hydroxyl groups of activated carbon and negatively charged sulfonic groups of RB5 is shown in Scheme 1 (interaction a).

**Figure 2:**
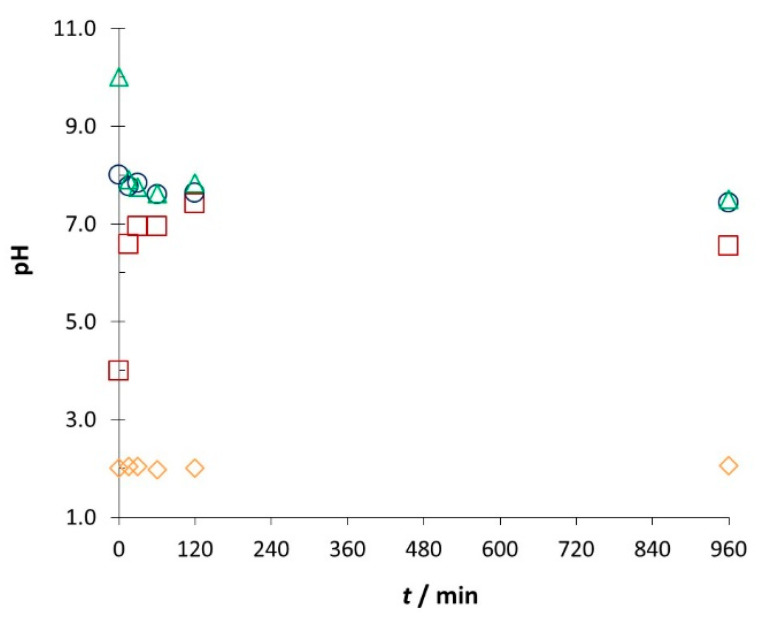
pH profiles of RB5 dye after adsorption for four initial pH values after appropriate time of adsorption (*t*) (**◇** pH_0_ = 2.00; **☐** pH_0_ = 4.00; **O** pH_0_ = 8.00; **△** pH_0_ = 10.00.

Throughout the whole adsorption time, pH values of dye solutions for pH_0_ = 2.00 were practically identical to the initial pH values (Figure 2). In contrast, for pH_0_ = 4.00, besides RB5 dye, the surface of activated carbon adsorbed more H^+^ ions than OH^−^ ions [27], and therefore, the pH value after adsorption increased already after 15 min; then, it gradually formed a plateau and stabilised at pH ≈ 6.5. For an initial pH value of pH_0_ = 8.00, the pH value after adsorption slightly decreased and stabilised at pH ≈ 7.4.

**Figure 3:**
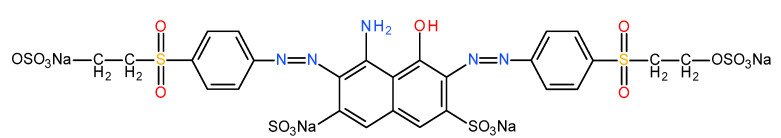
Chemical structure of Reactive Black 5 dye.

This indicates that electrostatic interactions at pH_0_ = 4.00 and pH_0_ = 8.00 are not the driving force of adsorption anymore. Therefore, it can be concluded that dye is most likely bound to the adsorbent by weaker interactions, hydrogen bonds, or/and van der Waals forces, which resulted in significantly smaller *E*_d_ values. Activated carbon carboxyl and phenolic groups may be responsible for the formation of hydrogen bonds with RB5 dye donors or acceptors groups, e.g., –NH_2_, –S=O, and −O−H [27]. Two examples, in which activated carbon and RB5 are donors and acceptors of hydrogen bonds, are presented in Scheme 1 (interactions b and c). The surface of activated carbon at pH_0_ = 10 is negatively charged, which can lead to possible rejection between the activated carbon surface and RB5 dye anion. Then, high *E*_d_ values at this pH initial value could be attributed to attractive π···π interactions [31]. The structure of the RB5 dye (Figure 3) contains four aromatic rings that allow its binding to the aromatic graphene layers of activated carbon by π···π aromatic interactions [32,33]. An example of such an interaction between the naphthalene ring of RB5 and graphene layer of activated carbon is shown in Scheme 1 (interaction d). These interactions, established between two parallel and mutually shifted aromatic systems at a distance of ca. 3.5 Å and with the interaction energy up to 50 kJ mol^−1^ [34], can also form RB5 multilayers on the surface of the activated carbon. Despite strong adsorption at pH_0_ = 10 observed in this study, it should be noted that the adsorption efficiency in most studies of RB5 adsorption on commercial powdered-activated carbon was still better in acidic media. The highest adsorption capacities were observed at pH values 2–4, which then decrease when the pH value increases to pH 6–7 [35,36,37,38].

### 2.2. Kinetics of Adsorption

In order to investigate the mechanism of adsorption, kinetic studies have been performed. Kinetic study is important to an adsorption process because it depicts the uptake rate of the adsorbate and controls the residual time of the whole adsorption process. The experimental data were analysed by three kinetic models: pseudo-first-order, pseudo-second-order, and intraparticle diffusion models. The pseudo-first-order and pseudo-second-order are the most often used models for the determination of kinetic parameters.

#### 2.2.1. Pseudo-First-Order and Pseudo-Second-Order Kinetic Models 

Lagergren [39] proposed a rate equation for the sorption of solute from a liquid solution based on the solid capacity. The kinetic model of this rate equation is expressed by following equation:

(1) $$\frac{dq_{t}}{dt}=k_{1}\left(q_{e}-q_{t}\right),$$

where *k*_1_ is the rate constant of pseudo-first-order (min^−^^1^).

Integrating this equation for the boundary conditions *t* = 0 to *t* = *t* and *q_t_* = *q_t_* gives a linear relationship expressed by the following equation:

(2) $$\ln\left(q_{e}-q_{t}\right)=\ln q_{e}-k_{1}\cdot t.$$

The pseudo-first-order kinetic constant *k*_1_ can be determined by plotting ln(*q_e_* – *q_t_*) vs. time (*t*), and if the pseudo-first-order equation is applicable, the plot should give a linear relationship with a high value of correlation coefficient (*R*^2^). The rate constant of pseudo-first-order (*k*_1_) can be calculated from the slope of this plot when the amount of adsorbed dye is at equilibrium (*q_e_*_,*calc*._) from the intercept.

Ho and McKay [40,41] developed a second-order equation based on adsorption capacity. This kinetic model is given by the following equation:

(3) $$\frac{dq_{t}}{dt}=k_{2}\left(q_{e}-q_{t}\right),$$

where *k*_2_ is the rate constant of pseudo-second-order (g mg^−1^ min^−^^1^).

Integrating this equation for the same boundary conditions as for the first-order gives the following equation in the linear form:

(4) $$\frac{t}{q_{t}}=\frac{1}{k_{2\;}\cdot q_{e}^{2}}+\frac{1}{q_{e}}\cdot t.$$

If the pseudo-second-order equation is applicable, the plot of *t*/*q_t_* against time (*t*) should give a linear relationship, and it allows the calculation of amount of adsorbed dye at equilibrium (*q_e_*_,*calc*._) from the slope and afterwards the rate constant of pseudo-second-order (*k*_2_) from the intercept. According to the pseudo-second-order model, as time approaches zero, the initial adsorption rate *h* (mg g^−^^1^ min^−^^1^) can be also calculated using the following equation [41,42]:

(5) $$h=\;\;k_{2\;}\cdot q_{e,calc.\;}^{2}.$$

The kinetic parameters for pseudo-first-order and pseudo-second-order models for all four initial pH values are given in Table 3.

The data in Table 3 show that values of the correlation coefficients (*R*^2^) for the pseudo-first-order model obtained from the linear plots defined by Equation (2) are relatively high, from 0.939 to 0.996. However, there is a great disagreement between the experimental (*q_e_*_,*exp*._) and calculated (*q_e_*_,*calc*._) values of the amount of adsorbed dye at equilibrium. This suggests that this adsorption system is not a first-order reaction and that possibly the pseudo-second-order model provides better correlation of the data.

Based on the pseudo-second-order model, the calculated *q*_e_ values (*q_e_*_,*calc*._) for all pH_0_ show much better agreement with experimental equilibrium values (*q_e_*_,*exp*._), while the values of the correlation coefficients are higher than 0.997 (Table 3). Therefore, it can be concluded that adsorption of RB5 dye on commercial activated carbon is kinetically controlled, assuming a pseudo-second-order rather than a pseudo-first-order process. As expected, a maximum *k*_2_ value was obtained for the pH initial value of pH_0_ = 2.00. As in the case of efficiency of decolouration (*E_d_*) values, the initial adsorption rate follows the same sequence: *h* (pH_0_ = 2.00) > *h* (pH_0_ = 10.00) > *h* (pH_0_ = 4.00 ≈ pH_0_ = 8.00).

#### 2.2.2. Intraparticle Diffusion Model

For evaluation of the diffusion mechanism, we also used the intraparticle diffusion model. Most adsorption processes involve three steps:

(i) Mass transfer of adsorbate from the solution to adsorbent surface,
(ii) Adsorption of adsorbate at a site on the surface of the adsorbent, and
(iii) Intraparticle diffusion of the adsorbate in the pores of adsorbent and adsorption at the site.

Step (ii) is often assumed to be very fast, and therefore, it cannot be treated as a rate-limiting step, while the adsorption of large molecules, for which longer contact time is needed to reach equilibrium, is almost always considered to be diffusion controlled by external film resistance and/or internal diffusion mass transport or intraparticle diffusion [27].

Theoretical treatments of intraparticle diffusion yield complex mathematical relationships, which differ in form as functions of the geometry of the adsorbent particle, and the intraparticle diffusion model could be based on the following equation [27,42]:

(6) $$q_{t}=k_{i}\cdot t^{0.5},$$

where *k*_i_ is the intraparticle diffusion rate constant (mg g^−^^1^ min^−^^0.5^).

**Figure 4:**
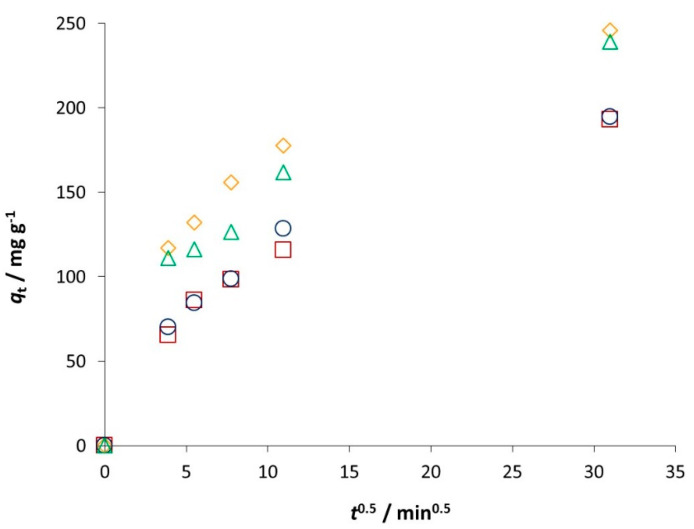
Root time plot for the adsorption of RB5 on activated carbon for four initial pH values (**◇** pH_0_ = 2.00; **☐** pH_0_ = 4.00; **O** pH_0_ = 8.00; **△** pH_0_ = 10.00).

If the intraparticle diffusion is a rate-limiting step of adsorption, i.e., if intraparticle diffusion controls the rate of adsorption, then plot *q_t_* vs. *t*^0.5^ should be linear and pass through the origin. If the plot presents multi-linearity, this indicates that intraparticle diffusion is not the only rate-controlling step and that two or more rate-controlling steps occur in the adsorption process [27,43]. Figure 4 shows the root time plots for the adsorption of RB5 onto commercial activated carbon for all initial pH values. All plots on this figure are not linear, i.e., they exhibit multi-linearity. Therefore, it could be concluded that intraparticle diffusion is not the only process that influences the adsorption rate and that multiple steps took place during the adsorption process.

### 2.3. Adsorption Thermodynamics

Thermodynamic adsorption parameters were calculated for the RB5 dye solution of initial pH value pH_0_ = 4. This pH value was chosen because it was the closest to the pH_0_ value of RB5 dye of the same concentration (pH_0_ = 4.83 [26]). Standard Gibbs free energy change values (Δ*G*°, kJ mol^−^^1^) of the adsorption process can be calculated from the equation:

(7) $$∆G^{o}=\;-R\;T\;\ln\;(K_{c}),$$

where *R* is the universal gas constant, and *T* is temperature. *K_c_* is the equilibrium constant calculated from the initial dye concentration (*c*_0_, mg dm^−^^3^) and the concentration of dye in the liquid phase at equilibrium (*c_e_*, mg dm^−^^3^) according to following equation [44]:

(8) $$K_{c}=\frac{c_{0}-c_{e}}{c_{e}}\;.$$

**Figure 5:**
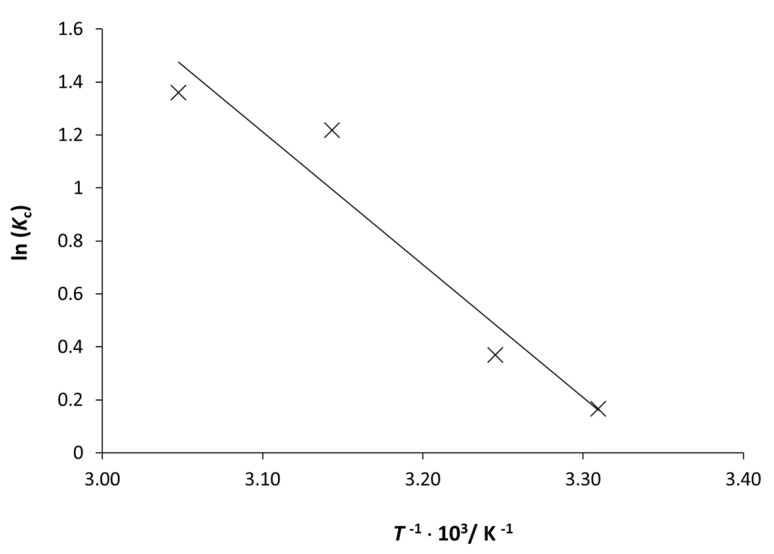
J. H. van’t Hoff plot for adsorption of RB5 dye on activated carbon at pH_0_ = 4.00.

Then, the values of the two other thermodynamic parameters were calculated from the van’t Hoff equation:

(9) $$\ln\;(K_{c})=-\frac{∆H^{o}}{R}\;\text{·}\frac{1}{T}+\frac{∆S^{o}}{R}\;,$$

where standard enthalpy change (Δ*H*°) can be calculated from the slope, and standard entropy change (Δ*S*°) can be calculated from the intercept of the plot ln(*K_c_*) vs. 1/*T*. The J. H. van’t Hoff plot resulted in a straight line with a correlation coefficient of 92.9% (Figure 5).

Table 4 presents the thermodynamic parameters (Δ*G*°, Δ*H*°, Δ*S*°) calculated from the experimental data by using Equations (7)–(9). The negative values of Δ*G*° show that the adsorption of RB5 onto commercial activated carbon was a spontaneous process whereby no energy input from outside of the system was required. As the higher negative value reflects a more energetically favourable adsorption, it can be concluded that adsorption at 328 K is energetically the most favourable. The positive value of Δ*H*° indicates the endothermic nature of adsorption process. As the adsorption process is usually exothermic, this phenomenon can be explained by the desorption of water molecules previously adsorbed on the dye molecule and the adsorption of dye molecules on the surface of the activated carbon [44].

Based on the values of standard enthalpy change, the adsorption mechanism can be concluded, i.e., the type of bond between adsorbent and adsorbate. Energies of moderate-strength hydrogen bonds, such as those in DNA/RNA, acids, and alcohols range from 16 to 60 kJ mol^−1^ [34]. Therefore, the ∆*H*° value for this adsorption system of ca. 42 kJ mol^−1^ indicates hydrogen bonds as a driving force for adsorption, which is in accordance with the previous discussion of adsorption at pH_0_ = 4.00 (Section 2.1). The positive value of Δ*S*° suggests the affinity of activated carbon for RB5 and increased randomness at the solid/liquid interface. During adsorption, the water coordinated molecules are displaced by dye molecules and consequently gain more translational entropy than is lost by dye molecules [29,43,44]. The data given in Table 4 also show an increase in *T*·Δ*S*° values with increasing temperature, and that *T*·Δ*S*° values are slightly bigger than the Δ*H*° value. This reveals that the adsorption process is a bit more dominated by entropic than enthalpic changes [45].

## 3. Materials and Methods

### 3.1. Chemicals

Reactive Black 5 (RB5) dye (Everzol Black B, supplied by Everlight Chemical Industrial Corp., Pineville, NC, USA, C.I. 20505, chemical formula: C_26_H_21_N_5_Na_4_O_19_S_6_, *M*_r_ = 991.82; purity = 75–80%, *w*/*w*) was used for the adsorption experiment. The chemical structure of the dye was prepared by the Biovia Draw program, Aachen, Germany. Powdered activated carbon was purchased from Kemika company, Croatia (particles size: <40 μm 85%, >80 μm 5%). Adsorbent was dried in an oven at 105 °C for 24 h and stored in desiccator until it is used.

### 3.2. Batch Mode Adsorption Studies

Adsorption studies were conducted by contacting 50 mL of RB5 dye solution of concentration *c*_0_ = 500 mg dm^−3^ at different initial pH values (pH_0_ = 2.00, 4.00, 8.00, and 10.00 adjusted by micro-additions of hydrochloric acid and sodium hydroxide solutions, *c* = 0.1 mol dm^−3^) with 0.1 g of powdered activated carbon in glass bottles. Suspensions were shaken at different contact times (15, 30, 60, 120 min, and 16 h until equilibrium is reached) with an impeller speed of 250 rpm at 45 (±1) °C (Heidolph Unimax 1010 with Incubator 1000). An adsorption time of 16 h was appropriate to reach adsorption equilibrium, as was determined in our previous study [23]. Adsorption experiments after 16 h were also performed at 29 (±1), 35 (±1), and 55 (±1) °C in order to determine the thermodynamic parameters of adsorption. All experiments were repeated three times under identical conditions to confirm the repeatability of the experiments. The experimental data in tables and points presented in figures are the average values of three repetitions. After agitation, suspensions were filtered through filter-paper blue ribbon, and the residual liquid-phase dye concentration after adsorption was determined spectrophotometrically by monitoring the absorbance using UV-Vis spectrophotometer (Lambda 20, Perkin Elmer, Cleveland, OH, USA) at a maximum absorbance wavelength (*λ*_max_ = 598 nm). The calibration graph of absorbance versus concentration obeyed a linear Beer–Lambert relation. For all filtrates, pH values after adsorption were also measured.

The amount of adsorbed dye at time *t*, *q_t_* (mg g^−^^1^), and at equilibrium, *q_e_* (mg g^−^^1^), were calculated by using following equation:

(10) $$q=\frac{V\;\cdot \left(c_{0}-c_{t}\right)}{m},$$

where *c*_0_ is initial dye concentration (*c*_0_ = 500 mg dm^−^^3^), *c_t_* is its concentration in the liquid phase at time *t* and at equilibrium (*t* = 16 h), *V* is the volume of liquid phase (dm^3^), and *m* is mass of the adsorbent (g).

The efficiency of adsorption, i.e., efficiency of decolouration (*E_d_*), is calculated by the following equation:

(11) $$E_{d}=\frac{\left(c_{0}-c_{t}\right)}{c_{0}}\times 100.$$

## 4. Conclusions

This study showed that the best adsorption results were obtained for initial pH values of pH_0_ = 2.00 and 10.00, which was presumably due to electrostatic interactions and π···π interactions, respectively. On the other side, at pH_0_ = 4.00 and 8.00, it can be assumed that RB5 dye is bound to the activated carbon by hydrogen bonds. This means that probably both chemical (electrostatic interactions) and physical adsorption (π···π interactions and hydrogen bonds) occurred depending on the initial pH values. Furthermore, it was shown that the adsorption of RB5 dye on commercial-activated carbon at all initial pH values is kinetically controlled, assuming a pseudo-second-order model, and that intraparticle diffusion is not the only process that influences the adsorption rate. Negative values of standard Gibbs free energy change indicate that the adsorption reaction is spontaneous in nature and that the adsorption of RB5 on activated carbon is energetically the most favourable at the highest temperature. A positive value of standard enthalpy change revealed the endothermic nature of the adsorption, while a positive value of standard entropy change suggests the increased randomness at the solid/liquid interface.

## Figures and Tables

**Table 1:** Amount of adsorbed Reactive Black 5 (RB5) dye (*q_t_*) at time *t* for different initial pH values.

*t*/ min	*q_t_*/mg g^−^^1^			
	pH_0_ = 2.00	pH_0_ = 4.00	pH_0_ = 8.00	pH_0_ = 10.00
15	117.1	65.5	70.5	111.0
30	132.0	86.0	84.7	116.4
60	155.9	98.5	99.0	126.7
120	177.9	115.8	128.5	161.7
960	246.0	193.0	194.9	239.1

**Table 2:** Efficiency of decolouration (*E_d_*) after appropriate time of adsorption (*t*) for different initial pH_0_ values.

*t*/ min	*E_d_*/%				
	pH_0_ = 2.00	pH_0_ = 4.00	pH_0_ = 8.00	pH_0_ = 10.00	pH_0_ = 4.83 [26]
15	46.8	26.2	28.2	44.4	-- ^a^
30	52.8	34.4	33.9	46.5	33.4
60	62.3	39.4	39.6	50.7	40.3
120	71.1	46.3	51.4	64.7	52.8
960	98.4	77.2	78.0	95.6	76.3

^a^ adsorption experiment was not performed.

**Table 3:** Kinetic parameters for RB5 dye adsorption on activated carbon for different initial pH values.

pH_0_	*q_e_*_,*exp*__._/ mg g^−1^	Pseudo-First-Order Model			Pseudo-Second-Order Model			
		*q_e_*_,*calc*._/ mg g^−1^	*R* ^2^	*k*_1_/ min^−1^	*q_e_*_,*calc*._/ mg g^−1^	*R* ^2^	*k*_2_/ g mg^−1^min^−1^	*h*/ mg g^−^^1^ min^−^^1^
2.00	246.0	136.6	0.9798	0.0060	256.4	0.9990	$1.169\cdot 10^{-4}$	7.69
4.00	193.0	127.9	0.9388	0.0044	204.1	0.9968	$8.745\cdot 10^{-5}$	3.64
8.00	194.9	134.3	0.9962	0.0058	204.1	0.9982	$9.656\cdot 10^{-5}$	4.02
10.00	239.1	142.2	0.9696	0.0049	250.0	0.9979	$9.222\cdot 10^{-5}$	5.76

**Table 4:** Thermodynamic parameters for the adsorption of RB5 dye on activated carbon for pH_0_ = 4.00.

*T*/ K	*c_e_*/ mg dm^3^	*c*_0_ − *c_e_*/ mg dm^−3^	*K*_c_	∆*G*°/ kJ mol^−1^	∆*H*°/ kJ mol^−1^	∆*S*°/ J mol^−1^ K^−1^	*T*·∆*S*°/ kJ mol^−1^
302	229.3	270.7	1.1805	−0.42	41.63	139.12	42.01
308	204.4	295.6	1.4462	−0.95			42.85
318	114.1	385.9	3.3821	−3.22			44.24
328	102.1	397.9	3.8972	−3.71			45.64

## Data Availability

Data are available in a publicly accessible repository.

## Sample Availability

Samples of the compounds are available from the authors.
